# Dental Civilization

**Published:** 1894-10

**Authors:** 


ARTICLE VII.

### DENTAL CIVILIZATION

BY A TRAVELED ENGLISH PRACTITIONER.

The progress of dental surgery since 1840 is remarkable The "grandeur of Rome and the glorious completeness of Grecian civilization,"as eulogized by Gibbon, resolves itself into mere play of words, when studied in the abstract. As practiced by the ancients, the art of dentistry was desultory, unscientific. The preservation of teeth by the brush and toothpick was taught from childhood. Fillings were rare being comparatively unknown till the second century B. C. Extractions were numerous. Diet figured largely in the prevention of caries. The indiscriminate use of acid and saccharine substances were unheard of, and while gorgeous banquets" were frequent in high circles, the regular table of the masses was simple—and, contrasted with the variety existing to-day, would be considered course.

The Phenicans, Greeks, Macedonians and Romans were extremely careful of their teeth, the latter taking advantage of the toothpick and brush, as used by their illustrious Greek neighbors. Toothpicks were made of silver, but preference given to the wood of the mastich tree. The Laws of the Twelve Tables* provided for "teeth bound with

---

*The laws of the Twelve Tables resulted from the unanimous voice of the Roman populace calling for some fixed and more appropriate code. In the year 454 B. C. ten ambassadors were sent to Greece to collect the laws of that polished people—principally those of Solon. These were embodied with others previously in force, and engraved on twelve tablets of brass—ten one year, two the next. The Twelve Tables were preserved in the temple of Jupiter Capitolinus. Nothing now remains but a few scattered fragments.

gold," it being lawful to burn or bury this gold with a dead person.

Artificial teeth have been known for centuries. Horace and Martialis speak of the custom as steadily growing. The charges, however, must have been exorbitant, the luxury being confined wholly to the nobility and wealthier classes. They were principally carved from bone, no system of coloring being employed to conform to original denture. Attempts are recorded of experiments by the Aztecs to apply the principles now in use, but with little success. Remains, however of retorts and crude moulds of clay fashioned to the shapes of ordinary molars have been unearthed. The Etruscan skull, found in 1885, has a few animal teeth arranged by gold bands in place of natural teeth. Various appliances for the relief of pain date back to the ninth century B. C.

It is comparatively of late years that dentistry has occupied anything like a properly recognized position among the different departments of minor surgery. For long it was practiced as an avocation, but not as a vocation, and without any professional education. Blacksmiths, barbers, watchmakers, and even itinerant peddlers were the tooth pullers. Even in some of our largest cities, these charlatans were, and still are, found practicing under the very shadow of universities and medical schools. It had not many attractions for medical men, and has not now.

The rapid strides of the dental profession in the United States is evidenced by the present number of dental colleges. America is far in advance of its foreign contemporaries. During my wanderings through Europe and a portion of Asia, the crying need of dental civilization was everywhere manifest. In the crowded hovels of Constantinople, Alexandria, Cairo, Jebba, Jaffa and Antioch, dentistry is almost unknown. American dentists are fairly well represented in Constantinople and the more civilized towns. In Athens, where centuries ago, Demetrius profaned the Parthenon by his debaucheries, and caused some of his maidens to undergo the indignity of sacrificing two central incisors for his amusement, dentistry is sadly neglected. Only foreigners and a few of the more intelligent natives seemed to take any care of their teeth.

Journeying into the interior, passing through Leopesi, Mandra, Daphni and Menidi, I found two dentists. Crossing from Attica to Morea, journeying southward through the towns of Cornith, Argos, Nauplia, Tripolitza, and Kalamata, the profession is poorly represented. Passing through the Suez, Great Britian on the right is fairly well represented; through the Red Sea into the Indian Ocean, touching at Bombay and journeying overland to Calcutta and northward to Delhi, dentistry is recognized as a dign fied professson. Good work is seen everywhere. The officers and families of H M. I. service, and many of the high caste natives, show evidence of dental care. The masses are particularly clean in this respect, but never visit a dentist until the last moment.

From India I jumped to China and was immediately shocked by the utter indifference to everything pertaining to even common dental care. Their diet is of such a nuture, however, that caries has been a national evil for centuries. Dentists—such as they are—being held in profound awe, are tolerated more as ornaments than necessities. The officers and representatives of the government, however, are proud of their "possession," and patronize hem as "luxuries." Japan is far more civilized in dental matters than her neighbor. At Yokohama a regular dental depot has been established for years. Superior work only is tolerated. Good prices prevail, and everything bears a prosperous aspect.

In the inland hamlets and towns of Russia dentistry is hardly known. St. Petersburg, Riga Vitebsk, Warsaw, Kharvok and the vicinity of Moscow support a fair representation of the profession. Great Britian writhes under spasmodic fits of dental civilization, and awakes from her lethargy about once every year to view the field and note the rapid transformation in surgical appliances. In everything pertaining to surgery, however, English surgeons are eminently inferior to any America has yet produced, and are only exceeded by France in celerity and professional aptitude. Three or four colleges devoted exclusively to dental science are established, and many of the medical colleges recognizing the necessity for special work, have attached a curriculum on dental work in all its branches. It is understood that no dental diploma is yet given by the Royal College of Surgeons in Scotland. Scotland has no licensing body, while diseases of the teeth and adjacent structures are made subjects of lecture and examination in the same manner as other regional and special diseases. A college of Dental Surgery is established at Edinburgh.

In conversation with a fellow traveler—a medical officer on H. M. S. "Camperdown"—he expressed surprise at the tendency of Americans to produce specialists in almost every branch of major and minor surgery and medical science, and hoped that "practitioners of dental surgery would restrict themselves to one branch of practice, be fully qualified medical men."

The restoration of order from the chaos of antagonism and scorn that enveloped dentistry fifty years ago, has been laborious and thankless. Each invention, till practitioners were educated to the manliness of sympathy, interest and toleration, was received with frowns and impressive contempt. The early promoters of dental civilization labored faithfully, ceaselessly: the mind kept ever before it the eventual emancipation of sufferers from the agonies of toothache, and the indiscriminate ravages of tooth extraction. They received little honor during life, but were overloaded when beyond the scene of mortal action. This system of carving the beneficient results of a faithful life on tombstones, with no substantial recognition during life, is barbarous.—*Items of Interest.*

